# Surgical Dyspepsia—A Case Report

**Published:** 1918

**Authors:** C. W. Roberts

**Affiliations:** Atlanta, Ga.; 402-403 Candler Building


### Journal-Record of Medicine

Successor to Atlanta Medical and Surgical Journal, Established 1855
and Southern Medical Record, Established 1870
**OWNED BY THE ATLANTA MEDICAL JOURNAL COMPANY**
PUBLISHED MONTHLY

*Official Organ Fulton County Medical Society, State Examing Board, Atlanta, Birmingham and Atlantic Railroad Surgeon's Association, Chattahoochee Valley Medical and Surgical Association, Etc.*

A. W. STIRLING, M. D., C. M., D. P. H.; J. S. HURT, B. Ph., M.D.
GEO. M. NILES, M. D., W. J. LOVE, M. D., (Ala.); Associate Editors

E. W. ALLEN, Business Manager

**COLLABORATORS**

W. F. WESTMORELAND, M. D., General Surgery
F. W. McRAE, M. D., Abdominal Surgery
ALLEN H. BUNCE, Medical Societies
E. B. BLOCK, M. D., Diseases of the Nervous System
MICHAEL HOKE, M. D., Orthopedic Surgery
CYRUS W. STRICKLER, M. D., Legal Medicine and Medical Legislation
E. C. DAVIS, A. B., M. D., Obstetrics
E. G. JONES, A. B., M. D., Gynecology
ALLEN H. BUNCE, M. D., Pathology and Bacteriology
R. T. DORSEY, Jr., B. S., M. D., Medicine
L. M. GAINES, A. B., M. D., Internal Medicine
GEO. C. MIZELL, M. D., Diseases of the Stomach and Intestines
L. B. CLARKE, M. D., Pediatrics
EDGAR PAULLIN, M. D., Opsonic Medicine
THEODORE TOEPEL, M. D., Mechano Therapy
E. G. BALLENGER, M. D., Diseases of the Genito-Urinary Organs

VOL. LXIV. ATLANTA, GA., MAR.-APR. 1918. NO. 12

### SURGICAL DYSPEPSIA—A CASE REPORT

**C. W. Roberts, M.D., Atlanta, Ga.**

I desire to invite your attention to the following Case Report, feeling that it is one of sufficient importance and interest to command your careful consideration. I have given the history and after treatment somewhat in detail, and if it proves tiresome to you to the extent of boring, I shall seek to re-instate myself in your good will by alluding to the fact that it is the lack of important detail in our current literature and text-books that makes us often search for help in vain, authors usually treating minute detail as of trifling importance.

W. D. N., Age 26, born in South Carolina, family history negative as effects this report.

Personal History: Had Typhoid Fever when sixteen years of age lasting some 10 weeks, from which he mad complete recovery. Soon after attack he weighed more than ever before, and was in perfect health.

Present trouble began five years ago as follows: After taking food, patient would have formation of gas on stomach and some two or three hours after meals would have extreme colicy pain in epigastric region of such severity as to require something for relief. Patient says he would take some soda or drink water or take some food and the pain would be relieved. This pain was accompanied by tenderness in the epigastrium, nausea, but no vomiting. Pain did not come after every meal but woul usually have at least one attack during each day. He rarely ever had attacks at night, and does not remember to have had an attack on getting up before breakfast.

Patient says that he consulted several physicians for this 'stomach trouble'' and when he took medicine and was careful about his diet, he would get better, so that there would be intervals of weeks or months when he was practically free from pain. After some three years of suffering of the above type, patient says he began to have pain radiate through to back about the region of the 11th or 12th dorsal verbetrae and around into left axillary region. Attacks were more severe and produced such nausea as to cause vomiting which would usually reileve the attack This vomiting was productive of only stomach contents making his teeth very sharp. Attacks came with the same irregularity but usually about two or three hours after a meal. After about a year of this type of suffering, during which time, under treatment and diet he would get better, to have a return of symptoms after intervals of partial relief, patient says all symptoms became exagerated, pain was moer constant and of such severity as to cause him to double up in bed, and would radiate all over upper abdomen; produced vomiting more often and his general health began to fail. His condition grew gradually worse until patient was vonfined to bed and for some three months previous to his admittance to Hospital he would vomit every day, after nearly quantityshrdluetaoinshrdl nshrdluetaoin shrdlu cmfw cmmm every meal, and became emaciated, losing some twenty-five or thirty pounds. On one occasion patient vomited a large quantity of clear blood and says that this was accompanied by extreme weakness. No historoy of dark or tarry stools.

On admittance to Hospital, patient presented the appearance of extreme emaciation, was very sallow, with hollow cheeks and eyes, very pale conjuntivae and was constantly eructating gas. Physical examination revealed nothing abnormal about chest, kidneys, genitals, or abdomen, save about epigastric region. On distention of stomach with a Seidlitz Powder, it was found greatly enlarged reaching below umbilicus an inch or more. Nothing could be palpated about the pylorus, but patient complained of tenderness about this region. Gall bladder area was negative. The first day in Hospital patient was given full diet, and he took al that was given ham because he was extremely hungry. There was no pain or vomiting until the second day when an attack came suddenly causing the vomiting of all solid food taken the day before, along with about one-half gallon of sour fluid mixed with mucus and an ocasional streak of blood. This vomited matter when left in a glass vessel for an hour showed the three layer formation seen in atonic dilitation of the stomach or retention of food from any cause; i. e., solid food at bottem, a clear area of liquid, and top layer of mucus. Chemical examination showed freed hydrochloric acid, and no lactic acid. Microscopic examination revealed an abundance of yeast cells. After vomiting the patient was relieved and ready for more food.

A Saltzer-Ewald test meal gave the following findings. Urine examination showed no albumin, sugar or bile. Haemoglobin was between fifty and sixty. Patient weightde one hundred and fifteen pounds.

Now, the history in this case and findings after admittance to Hospital, pointed very definitely to pyloric obstruction, from a healed or paritially healed ulcer. The sallow appearance of the patient led us to suspect ball bladder involvement, but careful examination showed no exidence of such complication, and on further questioning of the patient we learned that this was a family characteristic.

To sum up the history we have the following: A slowly developing stomach trouble with intervals of relief followed by another onset, and finally vomiting, causing temporary relief; then vomiting of blood. A chain of symptoms pointing pretty definitely to gastric ulcer. The findings after admittance to Hospital showed positively that the patient had pyloric obstruction and the test meals led us to exclude from the case the question of cancer as a cause| The finding of blood streaked mucus and a constant rather high free hydrochloric acid content argued in favor of an active ulcer.

Erproratory Laparotomy was advised and readily accepted. Under either the upper abdomen was opened through the right rectus, the gall bladder palpated and found free from adhesions, compressible, and without stones. On retracting the abdominal walls a large scar-like area involving the pyloric end of the stomach came into view. The entire ring of the pylorus was involved in a thick, rather hard mass, not permitting any penetrating of the pyloric opening. No active ulcer condition as evidenced by a crater-like feel in any part of this scar-like area could be made out; but the mass seemed simply an edematous infiltration of the pyloris with a very evident scar showing in the wall and etxending well around the stomach near the pyloric end. Several small glands were noted about the mass and in the meso-colon of the transverse colon.

Posterior Gastro-Enterostomy was decided upon as an operative measure for relief and rapidly performed by the suture method without clamps, making an anastometic opening of some two and one-half inches between the posterior wall of the stomach and the first part of the Jejunum.

Patient was put to bed in the Fowler sitting posture which was maintained throughout th econvalescent period. Normal saline proctoclysis begun at once and kept up at intervals for three days. Soon after being returned to bed patient vomited small quantity of dark fluid; this was the last vomiting, there being no more while in Hospital, nor any since being discharegd.

The onvalescent period was absolutely uneventful, patient being allowed warm water one ounce at a time the first night after operation, the quantity subsequently being gradually inreased. Liquid nourishment was given the second day and semi-solid diet the fifth day. At the end of the first week after operation patient was taking fairly full diet. On the morning of the third day, following a dose of castor oil the night before, patient had a good bowel movement containing considerable dark disorganized blood; a typical tarry stool. There were no abnormal stools after this. Wound healed primarily, and the patient was allowed out of bed on the eleventh day, and was discharged on the eighteenth day following operation.

There was absolute and immediate relief of all symptoms, with a gain of thirty pounds in two months.

Several months have elapsed since the operation and a recent report says, "I am perfectly well and am working daily on my farm."—402-403 Candler Building.

